# Education, Gender, and Cohort Fertility in the Nordic Countries

**DOI:** 10.1007/s10680-018-9492-2

**Published:** 2018-06-19

**Authors:** Marika Jalovaara, Gerda Neyer, Gunnar Andersson, Johan Dahlberg, Lars Dommermuth, Peter Fallesen, Trude Lappegård

**Affiliations:** 1 Department of Social Research, University of Turku, 20014 Turku, Finland; 2 Stockholm University, Stockholm, Sweden; 3 Statistics Norway, Oslo, Norway; 4 ROCKWOOL Foundation, Copenhagen, Denmark; 5 University of Oslo, Oslo, Norway

**Keywords:** Cohort fertility, Education, Gender, Finland, Denmark, Sweden, Norway

## Abstract

Systematic comparisons of fertility developments based on education, gender and country context are rare. Using harmonized register data, we compare cohort total fertility and ultimate childlessness by gender and educational attainment for cohorts born beginning in 1940 in four Nordic countries. Cohort fertility (CTF) initially declined in all four countries, although for cohorts born in the 1950s and later, the CTF remained stable or declined only modestly. Childlessness, which had been increasing, has plateaued in Denmark, Norway and Sweden. Women’s negative educational gradient in relation to total fertility has vanished, except in Finland, while men’s positive gradient has persisted. The highest level of men’s childlessness appears among the least educated. In the oldest female cohorts, childlessness was highest among the highly educated, but these patterns have changed over the cohorts as childlessness has increased among the low educated and remained relatively stable among higher educated women. In Denmark, Norway and Sweden, childlessness is now highest among the least educated women. We witness both a new gender similarity and persistent (among men) and new (among women) educational disparities in childbearing outcomes in the Nordic region. Overall, the number of low educated has decreased remarkably over time. These population segments face increasing social and economic disadvantages that are reflected as well in their patterns of family formation.

**Electronic supplementary material:**

The online version of this article (10.1007/s10680-018-9492-2) contains supplementary material, which is available to authorized users.

## Introduction

Over the past 60 years, European countries have experienced a remarkable expansion of education at the upper-secondary and tertiary levels. The proportion of persons lacking degrees beyond the basic level has halved within one generation. In 2015, only 15% of 25–34-year-olds in Europe (EU 22) were without post-basic education compared to 30% of 55–64-year-olds (OECD 2016, Table A1.3). This change has been particularly pronounced among women. In almost all post-industrial societies, young women now outnumber men in attaining tertiary degrees (Vincent-Lancrin 2008; Charles 2011).

A vast array of literature has documented the fundamental role of educational attainment in shaping women’s childbearing patterns, especially their timing of births and their childlessness. The findings generally reveal that highly educated women tend to have their first child at an older age and that their levels of childlessness are higher than those of less educated women (for reviews, see Hoem et al. 2006; Kravdal and Rindfuss 2008; Tanturri et al. 2015; Nitsche et al. 2015).

Little is known about whether and how the associations between women’s increasing educational attainments and their ultimate fertility have changed over time (van Bavel 2012; Tanturri et al. 2015). However, recent studies suggest that educational expansion has had only limited effects on patterns and developments in ultimate fertility (Sobotka et al. 2017) and childlessness (Beaujouan et al. 2016) by educational level in continental European countries. In most European countries, highly educated women are still more likely to remain childless than low-educated women (Wood et al. 2014; Miettinen et al. 2015; Tanturri et al. 2015).

Compared to research on women’s fertility, research on the fertility of men is scarce and almost uniformly suggests a positive association between men’s educational attainment and their fertility outcomes (Kravdal and Rindfuss 2008; Trimarchi and van Bavel 2017; Tanturri et al. 2015; Miettinen et al. 2015; Nisén et al. 2014, 2017; Nisén 2016). However, almost no research has addressed the ways in which increases in educational attainment and the reversal of the gender gap in education to women’s advantage are related to changes in men’s fertility outcomes.

When women regularly attain educational levels that are similar to or higher than those of men and economic and social roles become more similar, gender differences associated with education and fertility would also be expected to diminish. Studies examining the timing of entry into motherhood and fatherhood in France and women’s and men’s fertility postponement and childlessness in Britain have resulted in some support for this ‘convergence hypothesis’ (Winkler-Dworak and Toulemon 2007; Kneale and Joshi 2008). However, whether the gendered patterns in fertility may indeed converge is an open question. It is furthermore unclear whether differences in women’s and men’s fertility would converge within educational groups only or whether educational differences in fertility outcomes would also diminish. In the first case, we would find ‘convergence across gender lines’, while educational differences persist; in the latter case, we would find ‘convergence across educational lines’ and consequently diminishing differences in fertility outcomes between educational segments of both women and men.

This study examines the ‘convergence hypothesis’ in greater detail by differentiating between ‘convergence across gender lines’ and ‘convergence across educational lines’ in cohorts born since the 1940s in the Nordic countries. The Nordic welfare states are often considered to be forerunners in developing both social and gender equality. Therefore, when researching the ways in which gendered associations between education and fertility have developed over time, the Nordic countries may establish a blueprint for similar changes in other post-industrial societies. In the Nordic countries, the aim of social equality is to reduce differences between social strata (here represented by the level of education). The aim of gender equality is to foster equal participation of women and men in paid work, family care work, and society at large. Considering both core aims, equality is generally understood as ‘equality of outcome’ (Phillips 1999). The aim is to create a society in which social and gender differences are minimized. Thus, the Nordic countries have been forerunners in Western Europe in promoting formal education and establishing a largely unified educational system. They have also been forerunners in implementing policies that reduce the opportunity costs of family formation and support both sexes’ participation in paid employment and childrearing. Given these aims and policies, we might expect relatively small differences in fertility patterns between educational groups, between men and women, and between the Nordic countries. Indeed, previous research suggests that unlike in many other societies, educational differences in women’s completed cohort fertility in the Nordic countries have diminished over time (Andersson et al. 2009; Kravdal and Rindfuss 2008; Miettinen et al. 2015).

Our study compares ultimate fertility by gender and educational attainment for cohorts born in 1940–1967/69 for men and 1940–1972/74 for women in four Nordic countries: Denmark, Finland, Norway, and Sweden. In other words, we compare fertility differentials between (a) birth cohorts, (b) men and women, (c) educational segments, and (d) four Nordic countries. This allows us to follow the change in fertility patterns across educational and gender lines and to draw conclusions about the potential forces behind these developments.

This analysis focuses on two aspects of ultimate fertility: completed cohort fertility, or Cohort Total Fertility (CTF, mean ultimate number of children), and the levels of ultimate childlessness, measured at age 45 for men and age 40 for women. We harmonize longitudinal individual-level data drawn from the national population registers of the Nordic countries and use a straightforward cross-sectional approach of measuring education simultaneously with ultimate fertility (at age 45 or 40).

**Fig. 1:**
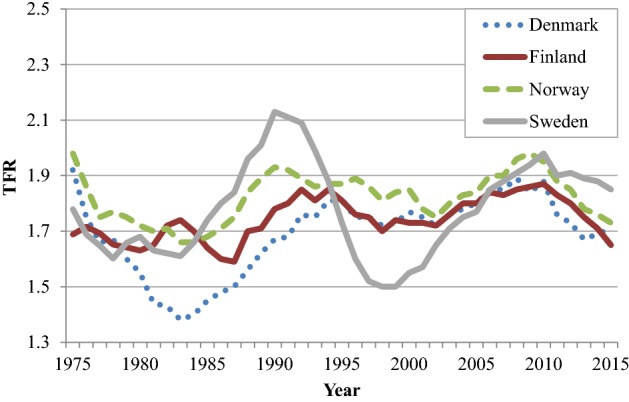
Period total fertility rate (TFR) in the Nordic countries, 1975–2015. *Source*: Nordic statistical central bureaus

We adopt a cohort approach. Period measures such as the period Total Fertility Rate (TFR) are otherwise common in official statistics. However, they mainly provide information on short-term changes in fertility behaviour. As a background, Fig. 1 shows the development of TFRs in the four Nordic countries during 1975–2015. The TFRs of each country show similar developments, with strong fluctuations in all countries (strongest in Sweden; see also Andersson 2000, 2004). Such fluctuations in the TFR are commonly due to changes in the timing of births, which does not affect the CTF.

Our analysis partly builds on a previous study by Andersson et al. (2009), which focused on the cohort fertility of women in the Nordic countries. A key contribution of the present study is that it also covers male cohorts, with the aim to produce a gender comparison of fertility patterns over time. The present study also covers more recent cohorts and developments. Finally, it uses a somewhat different approach than Andersson et al. (2009), which defined education as the attainment at age 30 and used a prospective cohort approach (see our methods discussion).

Our empirical analysis begins with an overview of the development of CTF and childlessness among men and women in each Nordic country. Then, we summarize the developments in educational attainment. Finally, the analysis focuses on the developments of educational differences in CTF and ultimate childlessness across cohorts, comparing women and men within and between the four countries. Before moving on to the data and empirical analysis, we give a condensed overview of prevalent theoretical perspectives on the relevant associations.

### The Association Between Education and Fertility: Modified by Gender, Institutional Context, and Educational Expansion?

Three theoretical approaches lend themselves particularly well to investigating how educational expansion and social change relate to fertility convergence across gender and educational lines: economic, gender equity, and institutional aspects. Microeconomic theories of fertility suggest that higher levels of socioeconomic resources positively influence couples’ childbearing but that this influence is also gendered with men’s resources having a more consistently positive effect (Becker 1993). In principle, both women and men contribute to the household’s economic resources, and greater resources add to the ability of the family to invest in having and raising children—referred to as the ‘income effect’.^1^ However, an opposing force ostensibly influences women: motherhood obligations lead to limitations in career opportunities and losses in work earnings, referred to as the opportunity costs of motherhood (Joshi 1990). Owing to their greater earnings potential, highly educated women are assumed to have higher opportunity costs and thus lower probabilities of childbearing than low-educated women. However, these arguments clearly rest on the assumption that women are the only or primary caregivers of their children and that they interrupt their employment to rear their children. By contrast, men are viewed as the main economic providers in a family, and fatherhood is not seen as conflicting with men’s employment and career; highly educated men are assumed to have higher fertility than low-educated men due to their greater capability to secure a sufficient living standard for their family.

Many scholars argue that these gendered associations are weakening or even reversing. This is mostly attributed to changing gender relations in the public and private spheres (McDonald 2000; Esping-Andersen and Billari 2015; Goldscheider et al. 2015; Anderson and Kohler 2015). Theories on gender and fertility development have suggested a decline in fertility with women’s rising participation in education and employment, which would make women dually burdened with paid work and care of their families. Scholars further argue that if men take more caring responsibilities and share family work more equally with their partners, fertility levels may be maintained or even increase (McDonald 2000; Neyer et al. 2013; Esping-Andersen and Billari 2015; Goldscheider et al. 2015; Anderson and Kohler 2015). All of these theories imply that greater gender equity within the family will have the strongest fertility-promoting effect among highly educated women because of their greater opportunity costs when their involvement in paid work is restricted.

Institutional approaches to fertility focus on the role that institutions and welfare states play in retaining or modifying gender, social, or economic relationships in society and in the family (Neyer 2003). These approaches assume that the institutional context shapes the links between gender, education and fertility (Neyer and Andersson 2008; Kravdal and Rindfuss 2008; Neyer et al. 2017). Social policies in the Nordic countries have often focused on supporting social and gender equality in areas relevant for family life. For example, the affordable and high-quality childcare that is available in the Nordic countries helps each parent in reconciling employment and childrearing and therefore reduces the opportunity costs of having a(nother) child. A positive role may also be played by parental leave policies that support the gender-equal division of care, facilitate the combination of work and care for both parents, and substitute reductions in work earnings through earnings-based benefits during family leaves. Gender- and social-equality measures incorporated into labour market and welfare policies may prevent direct or indirect disadvantages in terms of employment and career that result from parenthood. In the Nordic countries, these policies and measures have been in place since the late 1960s in order to increase women’s participation in the labour market, promote men’s participation in family work and care, and establish a society with gender equality. These policies are considered important factors in maintaining high fertility in the Nordic countries (Andersson et al. 2009).

In addition to educational expansion and the reversal of the gender gap in education, the labour-force participation of women has also increased, and their economic position has strengthened in society as well as in families. The relative contributions of partnered women and men to family incomes have changed; an increasing portion of partnered women have similar or higher education and earnings than their partner (Chudnovskaya 2017a; Klesment and van Bavel 2017; Mäenpää and Jalovaara 2015). The Nordic countries have been at the forefront of these developments. In the two-earner family model that now prevails in the Nordic countries, women and men are more similar than before in their economic and domestic roles, and both partners’ earnings are considered essential for the economic security of the family.

Taken together, developments in the private and public sphere lead us to suspect that in contemporary Nordic countries, women’s high education is no longer as much at odds with childbearing as it used to be and as conventional economic theories suggest. Theories on the relationship between education and fertility in post-industrial societies mainly concern the opportunity costs that discourage childbearing, particularly among highly educated women. However, as argued above, these opportunity costs may be less important in societies such as the Nordic ones in which the two-earner family model prevails and where women are able to combine childbearing and paid employment with relative ease. The opposing income effect could then be expected to gain in relative importance among women: having sufficient economic resources to sustain a family would promote childbearing not only among men but also among women (Andersson 2000). Having limited economic resources would have the opposite effect. The latter draws attention to the obstacles to childbearing among the low educated for both sexes. Unemployment risks are higher and earnings are lower among low-educated men and women, creating labour market insecurities that may be barriers to childbearing. Given that low-educated women are most likely to partner with low-educated men, these insecurities may accumulate in couples. Additionally, the lowest educated have the lowest likelihood of forming a union at all and of retaining it; in the Nordic countries, this applies to both genders (Bracher and Santow 1998; Jalovaara 2012, 2013). Therefore, the shorter time spent in stable partnerships is a factor that further links low education to reduced fertility (Trimarchi and van Bavel 2017). Moreover, the expansion of education may have aggravated the fertility-depressing conditions for lower educated women and men. With the general increase in education, the low educated have been crowded out of many segments of the labour market (Gautier et al. 2002; Albrecht et al. 2009). This process was intensified by the restructuring of economies from industrial to high-skilled post-industrial activities. These developments suggest that having children may have become more difficult for the low educated regardless of gender. In other words, we expect that the inverse association between women’s education and fertility has attenuated with the lower educated facing increasing obstacles to childbearing.

## Data and Methods

Our analyses are based on individual-level data drawn from population registers and from the registers of educational degrees of each Nordic country. The Nordic countries have a long history of full and reliable registration of their resident populations and their vital events. Residents of the Nordic countries have a unique personal identification number, which enables an accurate, computerized linking of information from different data sources. Our data cover the entire population of Denmark, Norway and Sweden as well as an 11% random sample of the population of Finland.

We follow the logic of a true birth-cohort design. We focus on women and men who were born in their respective Nordic country. Further, we only include data on persons who were alive and resided in their birth country at age 40 for women and age 45 for men. Age is measured at the end of the calendar year. Return migrants, i.e. persons who were born in the respective country, emigrated and had returned by age 40 (women) or 45 (men), are included in the analysis. Other migrants, that is, immigrants born abroad and emigrants who did not return to their birth country, are not included.

The analysis covers 5-year cohorts born from 1940 to 1974. We have complete cohort data for all individuals born in 1940–44 up to the 1960–64 5-year cohort. For the youngest cohorts (1965–74), the study populations vary by country and by sex due to the different age limits for women and men and the last year for which data are available. For women born in 1965–69, we have complete data for all four countries. For those born in 1970 onwards, we have a 5-year cohort coverage (1970–74) for Denmark and Norway and a 3-year cohort coverage (1970–72) for Finland and Sweden. Likewise, for men born in 1965–69, our study comprises complete 5-year cohorts for Denmark and Norway and 3-year cohorts for Finland and Sweden.^2^

The two outcomes of our study, completed cohort fertility and ultimate childlessness, are based on the dates of all recorded childbearing events (live births). Births to return migrants while they were abroad are also included, although some births may be missing (e.g. if the child remained or died abroad). Unlike in survey data on men in which births in previous unions and non-marital births in particular often go unreported (Rendall et al. 1999), the registers cover men’s childbearing histories nearly as completely as those of women. Nevertheless, some slight proportion of the lower cohort fertility and higher childlessness among men than among women likely reflects the fact that fatherhood is sometimes not registered.^3^

We measure cohort total fertility and childlessness at age 45 for men and 40 for women. A small portion of women and men have additional children beyond these ages,^4^ but choosing higher age limits would lead to the exclusion of the most recent cohorts and thus limit our opportunities to assess the latest trends in completed fertility and ultimate childlessness.

In our study, we measure education at the same age as ultimate fertility and childlessness are measured, that is, at age 40 for women and 45 for men. We chose this approach because it is simple and direct. To achieve maximum comparability across countries, we harmonized the definitions of educational level attained using the 1997 International Standard Classification of Education. The groups are:

**Table:** 

Low education (primary and lower secondary)	ISCED 0–2
Medium education (upper secondary)	ISCED 3–4
High education (tertiary)	ISCED 5–6

We excluded data on the very few cases with missing information on educational attainment. The exception is Finland, whose data do not allow us to distinguish persons with missing educational information from those with the lowest educational level. Although the data were maximally harmonized, the country comparisons may be somewhat hampered by differences and changes in educational systems, educational codings and classification procedures. However, the comparisons benefit from the fact that the Nordic educational systems are fairly similar and that most data differences do not affect the comparability of trends.

Compared to the study by Andersson et al. (2009), we have slightly different education data for Norway. For this country, we could apply a revised classification of education, which is better adjusted towards international standards (Statistics Norway 2006). Because of this revision, the proportion of low educated persons in Norway is lower and the proportion of medium educated is higher. The new classification is more in line with the standard of the other Nordic countries.

Methodologically, one central question is how and when to measure education and fertility. Education and childbearing are often interacting processes: education affects childbearing, but childbearing, especially at an early age, may also affect further education. Results on the association between education and fertility are therefore sensitive to when (at what age) and how (by which measure) education is measured (Hoem and Kreyenfeld 2006; Kravdal 2004), and the same applies to different methods of studying fertility outcomes. Our approach differs somewhat from that by Andersson et al. (2009), where education was measured at age 30 and a prospective study design was applied. In the Nordic countries, movements in and out of education occur frequently, and many people become parents prior to finishing their highest level of education (Hoem et al. 2006; Tesching 2012). Therefore, determining the ideal age to measure education is difficult. We explored alternative possibilities. Some cases resulted in minor differences compared to our study design and to the previous study by Andersson et al. (2009). Since the differences did not substantially affect the overall results and their interpretation, we settled for measuring educational attainment at the same age as when we measure fertility.

In what follows, we present our results as figure diagrams. The data are also made available as tabulations in the Electronic Supplementary Material.

## Results

### Fertility Developments: Cohort Total Fertility

**Fig. 2:**
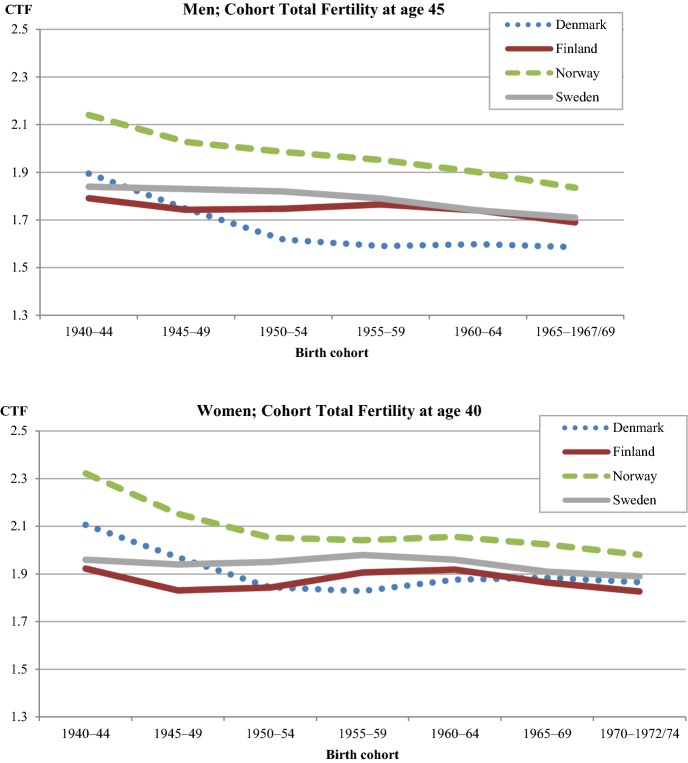
Cohort total fertility for men (at age 45) and women (at age 40) in 5-year cohorts born in each Nordic country, 1940–. *Source*: Nordic register data, authors’ own calculation

Figure 2 shows the development of cohort total fertility (CTF) for men and women in each Nordic country. CTF declined relatively little in the four countries. In contrast to the fluctuating developments in period TFRs (Fig. 1) and to the more pronounced or even dramatic declines of the CTFs in other European countries (Frejka 2017), cohort fertility has remained fairly stable in the Nordic region. In Denmark and Norway, cohort fertility fell somewhat among the oldest cohorts but remained fairly stable thereafter. The CTFs of Finland and Sweden have been at almost the same level across all cohorts.

Female CTFs have been consistently close to the replacement level in all countries and cohorts. Neither the very low period TFRs of Denmark (in 1983) and Sweden (in 1998–1999) nor the ‘roller-coaster’ fertility (Hoem 1990, 1993; Andersson 2000), which all Nordic countries experienced to some extent (see Fig. 1), had a resounding effect on CTF. This is partly because cohort fertility is more robust to changes in the timing of childbearing; it also reflects a recuperation of fertility at older ages. In the Nordic countries, the younger cohorts have their children at older ages, but on average, they have almost as many children as the cohorts born two to three decades earlier (Andersson et al. 2009). Differences between countries in women’s completed cohort fertility are small, and even further convergence of the CTFs has occurred among the most recent female cohorts.

In all countries, men’s completed fertility is somewhat lower than women’s, stemming from the fact that male birth cohorts are slightly larger than female birth cohorts. Among all cohorts of men born since the 1950s, Danish men have had the lowest CTF (1.6). This value has been close to the level that demographers regard as the threshold towards very low fertility for women (1.5, see Kohler et al. 2006). As with women, Norwegian men have had the highest completed fertility. Finland and Sweden fall between Denmark and Norway.

### Fertility Developments: Ultimate Childlessness

**Fig. 3:**
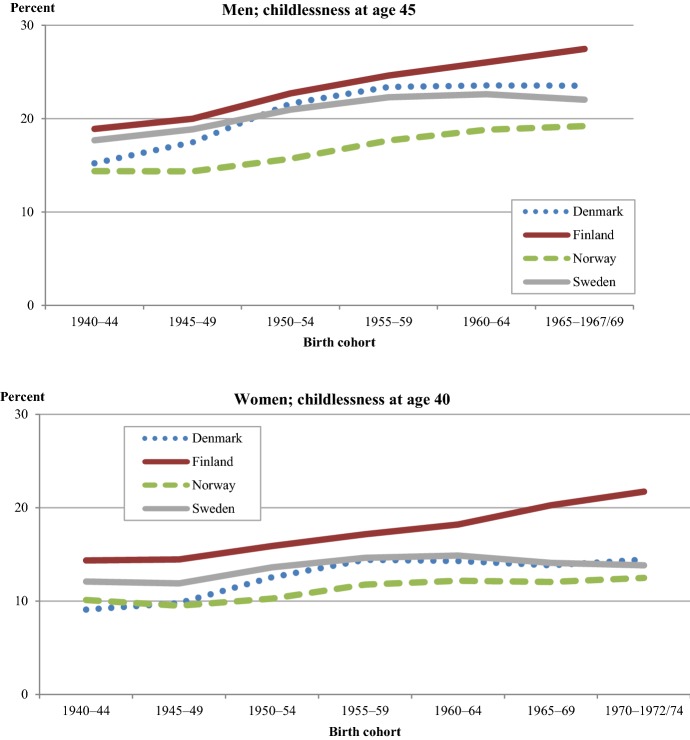
Childlessness (%) for men (at age 45) and women (at age 40) in 5-year cohorts born in each Nordic country, 1940–. *Source*: Nordic register data, authors’ own calculation

Figure 3 shows the cohort development of ultimate childlessness for men and women in each Nordic country. Compared to CTF, childlessness exhibits major differences between cohorts, between sexes, and between the countries. Ultimate childlessness has increased in all countries when compared to the level of the 1940s cohorts. However, in most countries and as far as women are concerned, the increase has been very moderate; it has plateaued with the most recent cohorts, and in these cohorts childlessness has even decreased slightly. Moreover, a small part of the observed increase in ultimate childlessness may be compensated by unobserved increases in childbearing beyond ages 40/45 (see footnote 4). The trend reversals in later cohorts with stabilizing or slightly declining levels of childlessness in Denmark, Norway, and Sweden, are not (yet) observable in Finland, where childlessness levels in the youngest cohorts are quite substantial: 27% for men and 21% for women (see also Sobotka 2017). Since cohort fertility has remained rather stable in Finland, the increase in childlessness has been counterbalanced by a simultaneous increase in higher-order childbearing. Norway had the lowest levels of childlessness across all cohorts of women and men, including the most recent one, with only 12% childless women and 19% childless men. Overall, the gender difference in ultimate childlessness amounts to some 6–10 percentage points.

### Developments in Educational Attainment

**Fig. 4:**
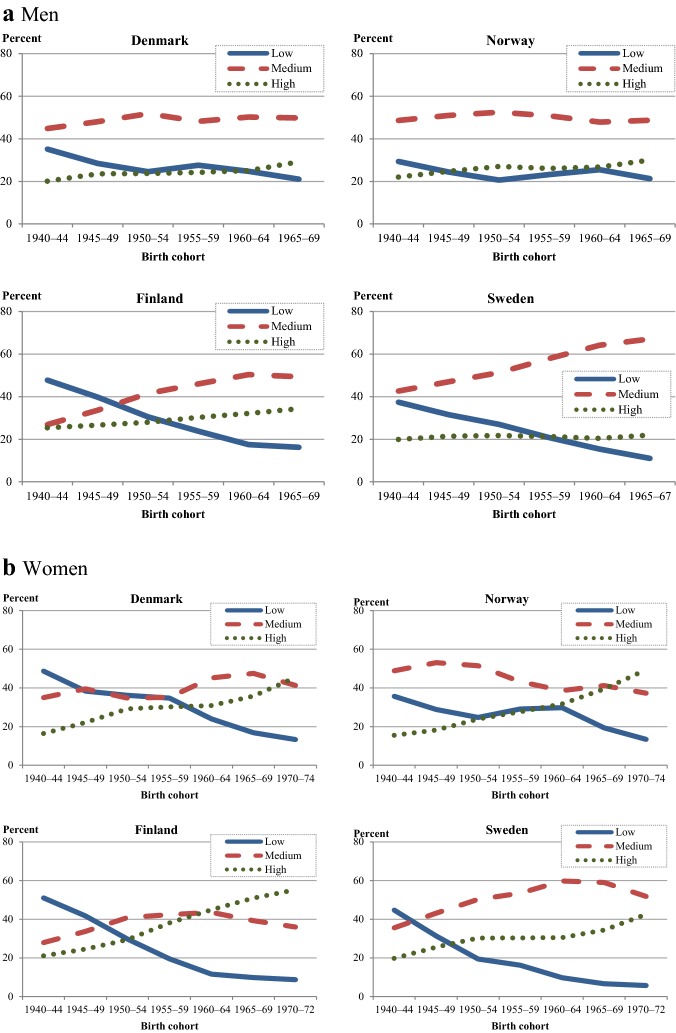
Educational level for men (at age 45) and women (at age 40) in 5-year cohorts born in each Nordic country, 1940–. **a** Men: educational level at age 45. **b** Women: educational level at age 40. *Source*: Nordic register data, authors’ own calculation

To provide background for assessing educational differentials in fertility development, we briefly describe the educational developments in each country. Figure 4 presents the percentages of men and women who have completed low (basic), medium (secondary), or high (tertiary) education as their highest educational level by cohort in each country.

Among men, the share of low educated has decreased across the study cohorts in all countries but most strongly in Finland and Sweden (− 32 and − 26 percentage points, respectively). In both countries, this has been paralleled by increases of almost similar magnitudes in the portion of men with medium educational attainment. In the other two countries, the percentage of men with medium education has only risen slightly (Denmark) or not at all (Norway). The increases in high educational attainment among men are rather modest (+ 8 to 9 percentage points) or, in Sweden, hardly existent (+ 2 percentage points).

Among women, the changes across cohorts have been stronger. The percentages of women with only a basic education have dropped markedly (22–42 percentage points). Among the oldest female cohorts, 36–51% of all women had only basic education; among the most recent cohort, the percentages varied between 6 and 13 (men: 11–21%). Medium educational attainment increased (except in Norway) with initial increases followed by slight decreases. In all countries, the share of women with high education has risen sharply. Between 43 and 55% of women born in the early 1970s held a tertiary educational degree compared to 16–21% of women born in 1940–44. As in many other European countries, women’s educational attainment exceeds that of men—the share of tertiary educated in our youngest cohort of men is 22–34%.

In sum, the low educated have become the smallest segment in both sexes. Women have clearly trended towards acquiring tertiary education, whereas among men, increases in tertiary education have been moderate, and medium education remains their most prevalent educational level by far. In older cohorts, men held higher educational degrees than women, but the secular developments have led to a reversal of the gender gap in education at both ends. In recent cohorts, the percentage of low educated is higher among men than among women, and the percentage of high educated is higher among women.

### Educational Differences in Cohort Total Fertility

**Fig. 5:**
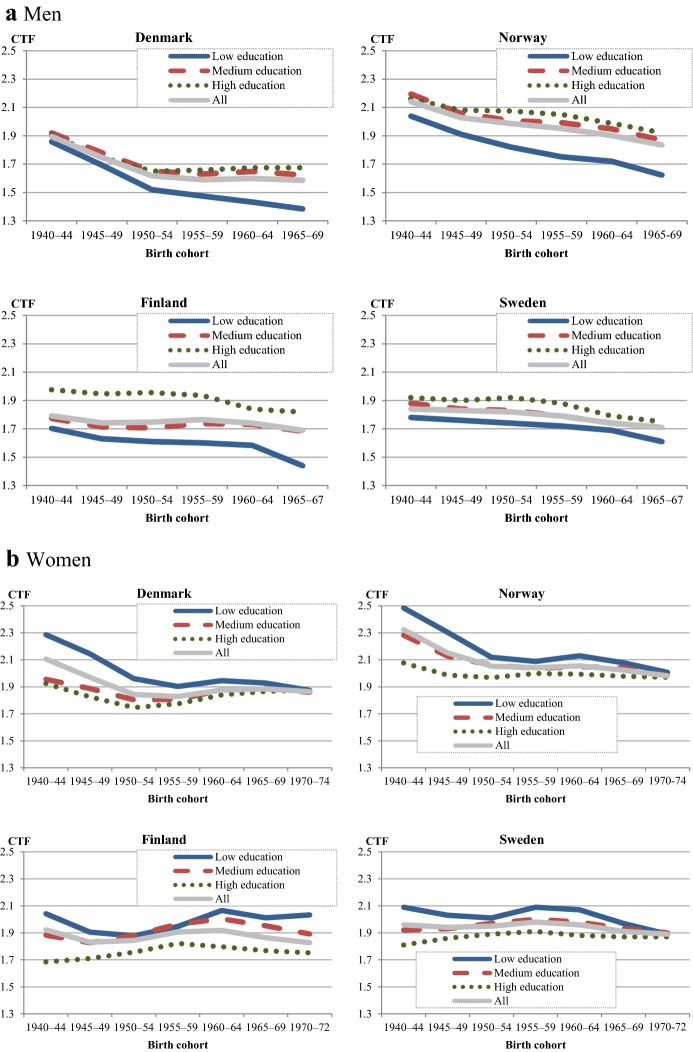
Cohort total fertility for men (at age 45) and women (at age 40) by educational attainment in 5-year cohorts in each Nordic country, 1940–. **a** Men: cohort total fertility at age 45. **b** Women: cohort total fertility at age 40. *Source*: Nordic register data, authors’ own calculation

Given the development of women’s and men’s educational attainment across cohorts, we examine whether concurrent changes can be discerned in cohort fertility based on educational segments. Figure 5 shows the development of educational differences in completed cohort fertility for women and men. Differences in cohort fertility by educational level are small in all Nordic countries, and patterns and developments are similar.

Across the male cohorts (Fig. 5a), CTF declined in all educational groups in all countries, although not to the same extent. Some decreases are negligible. These decreases differ from the stability in cohort fertility that we observed for the aggregate group of all men taken together (Fig. 2). These differences in trend developments are produced by compositional changes in which increasing fractions of men have received higher education. The CTF of low-educated men has consistently been the lowest. This holds across all countries and all cohorts. In Denmark and Norway, the difference between low-educated men and other educational groups of men has widened somewhat.

Female cohorts reveal a different pattern and development (Fig. 5b). The older cohorts in all countries display a result that is commonly reported in the literature: The lowest educated women had the highest completed fertility, and the highly educated women had the lowest. However, this pattern has almost completely vanished in the youngest cohorts. In Denmark, Norway, and Sweden, a remarkable convergence of CTF levels has occurred across cohorts; the differences between educational segments have practically disappeared. This is due to stronger declines in CTF among low-educated women and increases (Denmark) or stabilization (Norway and Sweden) among the higher educated. The Finnish pattern is different, where CTF has remained relatively stable across cohorts in all educational segments, and ultimate fertility is lowest among the highly educated and highest among the low educated also in the youngest cohorts of women. However, the differences are quite small, amounting to approximately 0.1 CTF unit per educational category.

A consequence of the main trends among men and women is that the gender gap in CTF by educational level has changed. Low-educated women of the older cohorts had the highest completed fertility, while low-educated men have had the lowest fertility in all cohorts. The decreasing fertility across cohorts among low-educated women led to the disappearance of strong gender differential patterns in all countries except Finland. Low-educated men still have a lower CTF than any other group, but low-educated women no longer stand out with elevated fertility.

### Educational Differences in Ultimate Childlessness

**Fig. 6:**
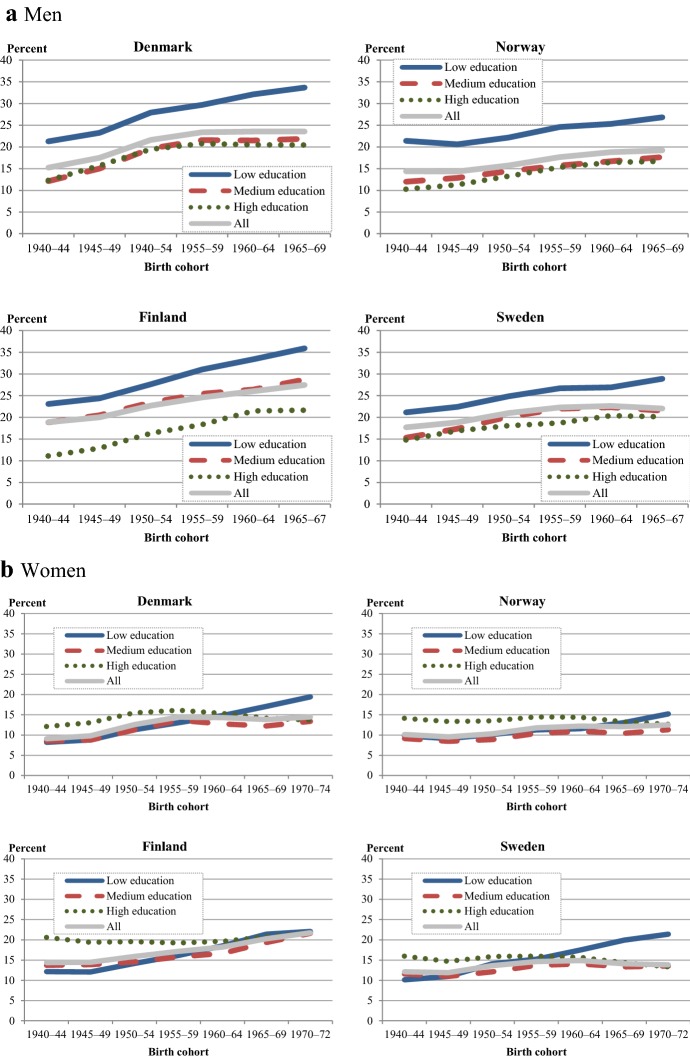
Childlessness (%) at age 45 (men) and 40 (women) by education in 5-year cohorts born in each Nordic country, 1940–. **a** Men: childlessness at age 45. **b** Women: childlessness at age 40. *Source*: Nordic register data, authors’ own calculation

In the previous sections, we observed that patterns and gradients in ultimate childlessness differed from those in ultimate fertility. Next, we present the developments of ultimate childlessness by educational attainment for women and men in the four Nordic countries (Fig. 6).

Men display remarkable differences in childlessness levels by educational attainment across all countries and cohorts (Fig. 6a). The lowest educated men have by far the highest levels of childlessness, while the differences in childlessness between medium and highly educated men are largely nonexistent (except in Finland). The striking difference in childlessness between men with basic education and those with higher levels has persisted across all cohorts in all countries. The gap has even increased somewhat as the increases in childlessness among medium and highly educated men have plateaued. Again, Finland deviates from this trend change. Among the youngest male cohorts, the difference in childlessness between the lowest and the highest educated men lies between 9 percentage points (Sweden) and 15 percentage points (Finland). Unlike in the other countries, Finland also has a clear difference in childlessness between men with medium and high educational attainment.

The levels of childlessness among men with only a basic education in the most recent cohorts are very high. More than a third (34–36%) of low-educated men born in 1965–69 in Denmark and Finland and more than a quarter (27–29%) of those born in Norway and Sweden were childless at age 45.^5^

The educational differences in childlessness and the development of those differences have been very different for women (Fig. 6b) than for men. In the oldest female cohorts in all countries, childlessness was clearly highest among the highly educated. It exceeded the levels of low-educated women by 4–9 percentage points. However, these patterns have changed entirely. In the most recent cohort, childlessness levels are highest among the lowest educated. The gap between the lowest and highest educated ranges from a narrow 1 percentage point in Finland to 8 percentage points in Sweden.

These developments have been similar in every country. Childlessness among low-educated women has increased almost continually, while the childlessness levels among medium and highly educated women have either remained stable, plateaued, or in the most recent cohorts, even slightly decreased. The gap in childlessness between highly and medium-educated women has narrowed across cohorts in all countries and has virtually vanished among the youngest female cohorts.

The stable or slightly decreasing levels of childlessness among highly educated women and the increasing levels of childlessness among highly educated men have also led to a reversal of the gender gap in childlessness among the highly educated in Finland, Norway, and Sweden. Among the older cohorts in these countries, highly educated women used to remain childless more often than highly educated men; among the younger cohorts, this pattern has changed. In more recent cohorts, highly educated women remain childless less often than highly educated men.

If we compare women’s and men’s development in childlessness across cohorts, we find an increase in gender similarity in childlessness patterns. This convergence has been driven by changes in ultimate childlessness with regard to the educational segments of women, while educational differences in men’s childlessness have not changed substantially. Meanwhile, we find an educational disparity in the younger cohorts that is increasingly similar for women and men. Overall, childlessness among the medium and highly educated seems to stabilize or even decrease, while childlessness among the low educated has risen. As a result, in the youngest cohort, childlessness levels are highest for the lowest educated, and this trend applies to men as well as women.

## Discussion and Conclusions

Using harmonized longitudinal individual-level data drawn from national population registers, we explored the development of ultimate cohort fertility and ultimate childlessness for cohorts born since the 1940s in four Nordic countries and compared the potentially gendered developments of educational differentials in fertility outcomes. In all four countries, the segment of persons with only a basic education has shrunk among both sexes. Among men, upper-secondary education is now the most common level attained, whereas among women, tertiary education has expanded substantially. These gendered developments have led to a reversal of the educational gender gap to women’s advantage. Comparing the development of ultimate fertility and childlessness across cohorts in the Nordic countries, we find similar trends in all countries, with the partial exception of Finland. The levels of ultimate fertility have remained fairly stable, especially in female cohorts born in the 1950s onwards, and differences between countries have effectively disappeared, especially with respect to female CTF. Childlessness increased in the early cohorts in all countries and more so among men than among women. In Finland, it increased across all cohorts.

The associations between gender and education in fertility outcomes have undergone fundamental changes across the cohorts. The developments are quite similar in the four countries. For men, educational fertility differentials have not changed substantially. In line with previous research on men’s fertility (e.g. Kravdal and Rindfuss 2008; Trimarchi and van Bavel 2017; Miettinen et al. 2015), we find that low-educated men have lower completed fertility and higher childlessness than medium or highly educated men. These disparities show a remarkable persistence across cohorts.

In contrast to men, women’s educational fertility differentials have changed substantively. Contrary to findings from many other European countries (Sobotka et al. 2017; Beaujouan et al. 2016), the negative educational gradients in CTF observed in older female cohorts have disappeared. Among women in the younger cohorts, no differences are discernible in ultimate fertility between educational categories of women in Denmark, Norway and Sweden.

Changes in women’s education-specific levels of childlessness are even more pronounced than the changes in the CTF levels. In all four countries, the childlessness of highly educated women remained fairly constant. Childlessness levels of medium-educated women converged towards those of the highly educated. In contrast, childlessness among low-educated women has been on an almost continual rise and has surpassed the levels of highly and medium-educated women. These differential trends have led to a reversal of the educational gap in childlessness among women in Denmark, Norway and Sweden. Highly educated women from the older cohorts had higher levels of childlessness than lower educated women, but in the youngest cohorts in Denmark, Norway and Sweden, the levels are highest among low educated women.

In Finland, we mainly observe a convergence in childlessness levels between different educational categories of women. In the case of CTF levels, slight differences in ultimate fertility by the educational categories remain. Evidently, the gendered patterns of fertility dynamics appear to evolve at a somewhat slower pace in Finland than in the other Nordic countries (for another similar example of differential change in gendered family dynamics, see Andersson et al. 2006).

The developments in women’s and men’s educational fertility differentials have brought about a new pattern of gender similarity in the Nordic region. The ‘convergence across gender lines’ in childlessness among the low educated is particularly noteworthy. In our oldest study cohorts, childlessness levels were highest for low-educated men and high-educated women, whereas in younger cohorts in all countries, childlessness is highest for the low educated regardless of gender. The new gender similarity accompanies a new ‘divergence across educational lines’ in fertility outcomes. The development of the youngest cohorts may well indicate that social inequality in childbearing is intensifying in that lower educated persons are increasingly left behind in family formation and that this applies to women and men equally. This, of course, goes hand in hand with the strongly diminished sizes of the low educated segments in each country.

Compared to the study by Andersson et al. (2009), including men and more recent cohorts provided novel insight into Nordic fertility dynamics and input to their potential explanation. Andersson et al. (2009) emphasized that welfare-state policies that focus on supporting gender equality may lead to a convergence in fertility across different educational groups. Extending their cohort series, we revealed that low educated women increasingly differ from the medium and higher educated. Adding analyses of male fertility allowed us to detect emerging gender similarities and persisting differences in completed fertility and childlessness.

How can we interpret these results in light of the outlined theoretical framework, namely, economic considerations, gender-equity assumptions, and institutional aspects, that links education and fertility? Our study can only provide hints about the numerous factors at play behind the developments we have observed. Our findings suggest that the fertility-stimulating effects of socioeconomic resources may have become more important for both women and men. If education is taken as a proxy for a person’s earnings potential, having a sufficiently high income or having the economic means to sustain a family may have become an increasingly important prerequisite for having (more) children for both women and men. The Nordic countries have numerous services and income transfers that should encourage childbearing among all social groups, but having children still appears to have become increasingly difficult for low-educated women and men. Additionally, the difficulties reconciling a career with family building that previously hampered the childbearing of highly educated women in particular seem to have been overcome.

We find some support for recent family-demographic gender theory and for the importance of institutional factors in shaping family-demographic outcomes. We assume that the gender-egalitarian ideology of the Nordic welfare regime, which originally targeted mainly women (Hernes 1987), contributed to halting increases in childlessness and declines in ultimate fertility. Institutional support for parents, particularly day care for children and support for mothers to remain in the labour force and for fathers to engage in childcare, eased the burden for mothers, countered the negative career consequences of motherhood, and reduced conflicts between women’s employment and family care. This orientation of the Nordic welfare regime seems to have mitigated the negative consequences of increased female education and labour-force participation on fertility.^6^

However, it is also noteworthy that even in the Nordic countries, some gender differences prevail. For instance, among men there is a clear positive education gradient in CTF, characterized by a large gap between the low- and middle-educated, while for women, the most prevailing pattern is a convergence in cohort fertility. Despite the fact that men and women in the Nordic countries are comparatively similar in their economic and domestic roles, differences prevail in gender norms, expectations and the potential to realize parenthood. In Nordic families, men are rarely the sole breadwinners, but their economic situation still seems to play a crucial role in family formation. Since there are more men than women in each cohort and women have higher education than men, it could well be that men who have less potential of being an economic provider (Chudnovskaya 2017b), who share (or can share) less in parenting (Duvander and Johansson 2014) or have other cognitive traits related to low education (Kolk and Barclay 2017) may find it increasingly difficult to form a family and have children.

Our findings have major implications for demographic research. They underline the need to carefully scrutinize the schemas of gender and social strata (here, education) that are applied in fertility research. As other researchers have also noted, we should be wary of the widely held notion that highly educated women necessarily have higher opportunity costs than low-educated women and that they therefore have fewer children than low-educated women (see also: Hoem et al. 2001; Andersson et al. 2009). This notion rests on the assumption that mothers are the sole caregivers of children and that motherhood and paid employment are strongly at odds, and it overlooks the value of available childcare, parental leave regulations, and partner’s engagement in childcare, all of which may modify the opportunity costs. In principle, the result may be that highly educated women have lower opportunity costs than the less educated (see Hoem et al. 2001; Kravdal and Rindfuss 2008). Overall, opportunity costs may not be as important as is usually assumed. Even if highly educated women have the largest earnings losses following from family leaves (Evertsson 2016; England et al. 2016), their remaining income may still be more than sufficient to maintain the family’s previous level of living, while the comparatively lower earnings loss of low-educated mothers may make it difficult for these women to make ends meet. Our research results clearly suggest the need to modify the existing economic concept of motherhood and to view women as breadwinners just as men are. The prevailing or even intensifying differences in CTF and childlessness between low-educated men and men with more education call for more thorough reflections and more stringent theoretical approaches to the links between changing gender relationships, manhood, fatherhood and men’s fertility (Hobson 2002).

The widening of the educational gap in childlessness for both women and men supports our presumption that fertility research should focus more attention on family-demographic developments among the low educated. With the increase in women’s and men’s educational attainment, the results indicate that the low educated have become an increasingly marginalized population segment not only with regard to their labour market position but also with regard to family formation. In the Nordic countries, affordable high-quality childcare has long been available to all children, and most fathers participate in childrearing. Therefore, the increase in childlessness among the lowest educated may not be readily explained by existing theories, such as gender theories on the division of work and care within the family. In particular, previous decline in marriage and increasing levels of union instability are other developments that may have hindered childbearing, particularly among the lower educated (Kravdal and Rindfuss 2008; Jalovaara and Fasang 2015, 2017). Research on the Nordic countries shows that the lowest educated men as well as women have the lowest likelihood of entering cohabitation and marriage, and the highest likelihood of union dissolution, and that stable employment promotes union formation and union stability regardless of gender (Bracher and Santow 1998; Jalovaara 2012, 2013; Jalovaara and Fasang 2015; Cooke et al. 2013). Indeed, one recent study suggests that the negative educational gradient in men’s childlessness operates chiefly via selection into union (Trimarchi and Van Bavel 2017). The importance of unions as mediating factors is largely missed by the theoretical discussion on the changing links between gender, education and fertility, as it focuses on couples. Overall, our research suggests that understanding fertility development in light of increased educational attainment and the reversal of the gender gap in education requires more thorough investigations of the demographic consequences of the increasing economic and social marginalization of low-educated women and men. Clearly, the currently prevailing demographic research perspective ‘from above’ needs to be complemented by a perspective ‘from below’.

Theories on men’s fertility highlight men’s position as family breadwinners and, more recently, as caregivers as well. Many researchers have noted the need to expand fertility research to include men (Bianchi 1998; Goldscheider and Kaufman 1996). Our research supports this view and further suggests that a gender comparison is essential. Our gender comparison of fertility developments allowed us to detect the increasing gender similarity, the increasing childlessness among the low educated that embraces both men and women, and the profound change in the link between education and fertility among Nordic women but not among men. It also demonstrated that despite these fundamental changes there are consistent patterns of gender differences which call for further investigation and explanation. Our research suggests that empirical and theoretical efforts to illuminate men’s fertility behaviour and the intersection of gender and social strata should not concentrate only on men but should use a gendered perspective.

## Electronic supplementary material

Below is the link to the electronic supplementary material.

Supplementary material 1 (DOCX 48 kb)

## Footnotes

1 A usual assumption is that the fertility-promoting ‘income effect’ may to some extent be counterbalanced by expectations on the amount of resources put on each child, i.e., investments in the ‘quality’ of children rather than their quantity (Becker 1974; see also Kravdal and Rindfuss 2008).

2 Comparative analyses between 3- and 5-year cohorts for Denmark and Norway showed no differences in results, so we expect that neither our comparison over time nor across countries is distorted by having only three-year cohorts for the most recent birth cohorts of Finland and Sweden.

3 Less than one percent of children born in Sweden in 1960 onwards had no registered father; for children born since 1980, the share was less than 0.5% (Statistics Sweden 2011). For Finland, our data show that about two percent of all registered births to mothers included no registered father. The share was highest in the oldest study cohort (1940–44: 3.4%) and less than 1.6% in cohorts of mothers born in 1955 and later.

4 The addition of ages 46–50 for recent cohorts of men would add about 0.03–0.04 child units to their CTF statistic. For women, the addition of ages 41–45 would add about 0.04–0.05 child units. The reduction of childlessness would be about 0.5–0.6 percentage points for men and 0.9–1.0 percentage points for women. The impact is most visible for the category of highly educated women: this category would add about 0.06 child units to its CTF statistic and reduce its childlessness statistic with about 1.4–1.5 percentage points.

5 This pattern does not change much if we consider the impact of any non-registration of fathers. Even if we assume that all non-registered fathers would belong to our study population of men being born and living in the country at age 45, childlessness among low educated men would only be slightly lower. Since non-registration of fathers was higher among the older cohorts than among the more recent ones, the increase in childlessness among men over time might have been even more pronounced.

6 Some of these changes have historical predecessors. Sandström (2014, 2017) reports on rapidly diminishing educational differences in the nuptiality and fertility of women in Sweden during the mid-twentieth century.
