# Supplementary material for: Education, Gender, and Cohort Fertility in the Nordic Countries
Source: Eur J Popul. 2018 Jun 19;35(3):563–86. doi: 10.1007/s10680-018-9492-2 (PMC6639448; doi:10.1007/s10680-018-9492-2)
Supplement: Supplementary file 1 — Supplementary material 1 (DOCX 48 kb) [file 10680_2018_9492_MOESM1_ESM.docx]

Online Supplementary Material: Tabulations

| Table 1. Cohort Total Fertility for men (at age 45) and women (at age 40), in five-year cohorts born in each Nordic country 1940–. | | | | | | |  |  |  |  |  |
| --- | --- | --- | --- | --- | --- | --- | --- | --- | --- | --- | --- |
|  | | | | | | |  |  |  |  |  |
|  | | | | | | |  |  |  |  |  |
| Men: CTF at age 45 | | | | | | | |  |  |  |  |
| Cohort | Denmark | Finland | Norway | Sweden |  |  |  |  |  |  |  |
| 1940–44 | 1.90 | 1.79 | 2.14 | 1.84 |  |  |  |  |  |  |  |
| 1945–49 | 1.75 | 1.74 | 2.03 | 1.83 |  |  |  |  |  |  |  |
| 1950–54 | 1.62 | 1.75 | 1.99 | 1.82 |  |  |  |  |  |  |  |
| 1955–59 | 1.59 | 1.76 | 1.95 | 1.79 |  |  |  |  |  |  |  |
| 1960–64 | 1.60 | 1.74 | 1.90 | 1.74 |  |  |  |  |  |  |  |
| 1965–67/69^1^ | 1.59 | 1.69 | 1.84 | 1.71 |  |  |  |  |  |  |  |
|  |  |  |  |  |  |  |  |  |  |  |  |
|  |  |  |  |  |  |  |  |  |  |  |  |
| Women: CTF at age 40 | |  |  |  |  |  |  |  |  |  |  |
|  |  |  |  |  |  |  |  |  |  |  |  |
| Cohort | Denmark | Finland | Norway | Sweden |  |  |  |  |  |  |  |
| 1940–44 | 2.11 | 1.92 | 2.32 | 1.96 |  |  |  |  |  |  |  |
| 1945–49 | 1.97 | 1.83 | 2.15 | 1.94 |  |  |  |  |  |  |  |
| 1950–54 | 1.84 | 1.84 | 2.05 | 1.95 |  |  |  |  |  |  |  |
| 1955–59 | 1.83 | 1.91 | 2.04 | 1.98 |  |  |  |  |  |  |  |
| 1960–64 | 1.88 | 1.92 | 2.06 | 1.96 |  |  |  |  |  |  |  |
| 1965–69 | 1.88 | 1.86 | 2.02 | 1.91 |  |  |  |  |  |  |  |
| 1970–1972/74^2^ | 1.87 | 1.83 | 1.98 | 1.89 |  |  |  |  |  |  |  |
|  | | | | | | |  |  |  |  |  |
| ^1^ 1965–67 for Finland and Sweden; 1965-69 for Denmark and Norway. | | | | | | | | | | | |
| ^2^ 1970–1972 for Finland and Sweden; 1970–1974 for Denmark and Norway. | | | | | | | | | | | |

Table 2. Childlessness (%) for men (at age 45) and women (at age 40) in five-year cohorts born in each Nordic country 1940–.

|  |  |  |  |  |  |
| --- | --- | --- | --- | --- | --- |
| Men: Childless at age 45 | |  |  |  |  |
|  |  |  |  |  |  |
| Cohort | Denmark | Finland | Norway | Sweden |  |
| 1940–44 | 15 | 19 | 14 | 18 |  |
| 1945–49 | 17 | 20 | 14 | 19 |  |
| 1950–54 | 22 | 23 | 16 | 21 |  |
| 1955–59 | 23 | 25 | 18 | 22 |  |
| 1960–64 | 24 | 26 | 19 | 23 |  |
| 1965–1967/69^1^ | 24 | 27 | 19 | 22 |  |
|  |  |  |  |  |  |
| Women: Childless at age 40 | |  |  |  |  |
|  |  |  |  |  |  |
| Cohort | Denmark | Finland | Norway | Sweden |  |
| 1940–44 | 9 | 14 | 10 | 12 |  |
| 1945–49 | 10 | 14 | 10 | 12 |  |
| 1950–54 | 13 | 16 | 10 | 14 |  |
| 1955–59 | 14 | 17 | 12 | 15 |  |
| 1960–64 | 14 | 18 | 12 | 15 |  |
| 1965–69 | 14 | 20 | 12 | 14 |  |
| 1970–1972/74^2^ | 14 | 21 | 12 | 14 |  |
|  |  |  |  |  |  |
| ^1^ 1965–67 for Finland and Sweden; 1965-69 for Denmark and Norway. | | | | | |
| ^2^ 1970–1972 for Finland and Sweden; 1970–1974 for Denmark and Norway. | | | | | |

Table 3. Educational level for men (at age 45) and women (at age 40) in five-year cohorts born in each Nordic country 1940–.

| a) Men: education at age 45 | |  |  |  |  |  |
| --- | --- | --- | --- | --- | --- | --- |
|  |  |  |  |  |  |  |
| Denmark |  |  |  |  |  |  |
|  | Cohort |  |  |  |  |  |
| Education | 1940–44 | 1945–49 | 1950–54 | 1955–59 | 1960–64 | 1965–69 |
| Low | 35 | 28 | 25 | 28 | 25 | 21 |
| Medium | 45 | 48 | 52 | 48 | 50 | 50 |
| High | 20 | 24 | 24 | 24 | 25 | 29 |
| Total | 100 | 100 | 100 | 100 | 100 | 100 |
|  |  |  |  |  |  |  |
| Finland |  |  |  |  |  |  |
|  | Cohort |  |  |  |  |  |
| Education | 1940–44 | 1945–49 | 1950–54 | 1955–59 | 1960–64 | 1965–69 |
| Low | 48 | 40 | 31 | 24 | 17 | 16 |
| Medium | 27 | 34 | 42 | 46 | 50 | 49 |
| High | 25 | 27 | 28 | 30 | 32 | 34 |
| Total | 100 | 100 | 100 | 100 | 100 | 100 |
|  |  |  |  |  |  |  |
| Norway |  |  |  |  |  |  |
|  | Cohort |  |  |  |  |  |
| Education | 1940–44 | 1945–49 | 1950–54 | 1955–59 | 1960–64 | 1965–69 |
| Low | 29 | 24 | 21 | 23 | 25 | 21 |
| Medium | 49 | 51 | 52 | 51 | 48 | 49 |
| High | 22 | 25 | 27 | 26 | 27 | 30 |
| Total | 100 | 100 | 100 | 100 | 100 | 100 |
|  |  |  |  |  |  |  |
| Sweden |  |  |  |  |  |  |
|  | Cohort |  |  |  |  |  |
| Education | 1940–44 | 1945–49 | 1950–54 | 1955–59 | 1960–64 | 1965–67 |
| Low | 37 | 32 | 27 | 21 | 15 | 11 |
| Medium | 43 | 47 | 51 | 58 | 64 | 67 |
| High | 20 | 21 | 22 | 21 | 20 | 22 |
| Total | 100 | 100 | 100 | 100 | 100 | 100 |

| b) Women: education at age 40 | | |  |  |  |  |  |
| --- | --- | --- | --- | --- | --- | --- | --- |
|  |  |  |  |  |  |  |  |
| Denmark |  |  |  |  |  |  |  |
|  | Cohort |  |  |  |  |  |  |
| Education | 1940–44 | 1945–49 | 1950–54 | 1955–59 | 1960–64 | 1965–69 | 1970–74 |
| Low | 49 | 38 | 36 | 35 | 24 | 17 | 13 |
| Medium | 35 | 40 | 35 | 35 | 45 | 47 | 41 |
| High | 16 | 22 | 29 | 30 | 31 | 36 | 45 |
| Total | 100 | 100 | 100 | 100 | 100 | 100 | 100 |
|  |  |  |  |  |  |  |  |
| Finland |  |  |  |  |  |  |  |
|  | Cohort |  |  |  |  |  |  |
| Education | 1940–44 | 1945–49 | 1950–54 | 1955–59 | 1960–64 | 1965–69 | 1970–72 |
| Low | 51 | 42 | 30 | 20 | 12 | 10 | 9 |
| Medium | 28 | 34 | 41 | 42 | 44 | 39 | 36 |
| High | 21 | 25 | 29 | 38 | 45 | 51 | 55 |
| Total | 100 | 100 | 100 | 100 | 100 | 100 | 100 |
|  |  |  |  |  |  |  |  |
| Norway |  |  |  |  |  |  |  |
|  | Cohort |  |  |  |  |  |  |
| Education | 1940–44 | 1945–49 | 1950–54 | 1955–59 | 1960–64 | 1965–69 | 1970–74 |
| Low | 36 | 29 | 25 | 29 | 30 | 19 | 13 |
| Medium | 49 | 53 | 51 | 43 | 39 | 41 | 37 |
| High | 15 | 18 | 24 | 28 | 32 | 39 | 49 |
| Total | 100 | 100 | 100 | 100 | 100 | 100 | 100 |
|  |  |  |  |  |  |  |  |
| Sweden |  |  |  |  |  |  |  |
|  | Cohort |  |  |  |  |  |  |
| Education | 1940–44 | 1945–49 | 1950–54 | 1955–59 | 1960–64 | 1965–69 | 1970–72 |
| Low | 45 | 31 | 19 | 16 | 10 | 7 | 6 |
| Medium | 36 | 43 | 50 | 53 | 60 | 59 | 52 |
| High | 20 | 26 | 30 | 30 | 31 | 34 | 43 |
| Total | 100 | 100 | 100 | 100 | 100 | 100 | 100 |

Table 4. Cohort Total Fertility for men (at age 45) and women (at age 40) by educational

attainment in five-year cohorts born in each Nordic country 1940–.

| a) Men: CTF at age 45 | | | | |  | | |  | b) Women: CTF at age 40 | | | | | | |
| --- | --- | --- | --- | --- | --- | --- | --- | --- | --- | --- | --- | --- | --- | --- | --- |
|  |  | |  | |  | | |  |  | |  | |  | | |
| Denmark |  |  | |  | |  | Denmark | | |  | |  | |  |  |
|  | Education | | | | |  |  | | | Education | | | |  |  |
| Cohort | Low | Medium | | High | |  | Cohort | | | Low | | Medium | | High |  |
| 1940–44 | 1.86 | 1.92 | | 1.91 | |  | 1940–44 | | | 2.29 | | 1.96 | | 1.92 |  |
| 1945–49 | 1.69 | 1.78 | | 1.75 | |  | 1945–49 | | | 2.15 | | 1.89 | | 1.83 |  |
| 1950–54 | 1.52 | 1.65 | | 1.65 | |  | 1950–54 | | | 1.96 | | 1.80 | | 1.75 |  |
| 1955–59 | 1.47 | 1.63 | | 1.66 | |  | 1955–59 | | | 1.90 | | 1.81 | | 1.77 |  |
| 1960–64 | 1.43 | 1.65 | | 1.67 | |  | 1960–64 | | | 1.95 | | 1.87 | | 1.84 |  |
| 1965–69 | 1.39 | 1.62 | | 1.67 | |  | 1965–69 | | | 1.93 | | 1.89 | | 1.87 |  |
|  |  |  | |  | |  | 1970–74 | | | 1.87 | | 1.86 | | 1.87 |  |

| Finland |  |  |  |  | Finland |  |  |  |
| --- | --- | --- | --- | --- | --- | --- | --- | --- |
|  | Education | |  |  |  | Education | |  |
| Cohort | Low | Medium | High |  | Cohort | Low | Medium | High |
| 1940–44 | 1.70 | 1.77 | 1.98 |  | 1940–44 | 2.05 | 1.94 | 1.72 |
| 1945–49 | 1.63 | 1.71 | 1.95 |  | 1945–49 | 1.92 | 1.88 | 1.75 |
| 1950–54 | 1.61 | 1.71 | 1.96 |  | 1950–54 | 1.89 | 1.91 | 1.82 |
| 1955–59 | 1.60 | 1.74 | 1.94 |  | 1955–59 | 1.96 | 2.00 | 1.87 |
| 1960–64 | 1.58 | 1.73 | 1.84 |  | 1960–64 | 2.06 | 2.05 | 1.86 |
| 1965–67 | 1.44 | 1.68 | 1.82 |  | 1965–69 | 2.01 | 1.99 | 1.83 |
|  |  |  |  |  | 1970–72 | 2.05 | 1.91 | 1.77 |
|  |  |  |  |  |  |  |  |  |
| Norway |  |  |  |  | Norway |  |  |  |
|  | Education | |  |  |  | Education | |  |
| Cohort | Low | Medium | High |  | Cohort | Low | Medium | High |
| 1940–44 | 2.04 | 2.19 | 2.16 |  | 1940–44 | 2.48 | 2.28 | 2.08 |
| 1945–49 | 1.91 | 2.06 | 2.08 |  | 1945–49 | 2.30 | 2.13 | 1.99 |
| 1950–54 | 1.82 | 2.01 | 2.07 |  | 1950–54 | 2.12 | 2.06 | 1.97 |
| 1955–59 | 1.75 | 1.99 | 2.05 |  | 1955–59 | 2.09 | 2.04 | 2.00 |
| 1960–64 | 1.72 | 1.95 | 1.99 |  | 1960–64 | 2.13 | 2.05 | 1.99 |
| 1965–69 | 1.62 | 1.87 | 1.92 |  | 1965–69 | 2.08 | 2.04 | 1.98 |
|  |  |  |  |  | 1970–74 | 2.01 | 1.98 | 1.97 |

| Sweden |  |  |  |  | Sweden |  |  |  |
| --- | --- | --- | --- | --- | --- | --- | --- | --- |
|  | Education | |  |  |  | Education | |  |
| Cohort | Low | Medium | High |  | Cohort | Low | Medium | High |
| 1940–44 | 1.78 | 1.88 | 1.92 |  | 1940–44 | 2.09 | 1.92 | 1.81 |
| 1945–49 | 1.76 | 1.84 | 1.90 |  | 1945–49 | 2.03 | 1.93 | 1.86 |
| 1950–54 | 1.74 | 1.83 | 1.92 |  | 1950–54 | 2.01 | 1.97 | 1.89 |
| 1955–59 | 1.72 | 1.79 | 1.88 |  | 1955–59 | 2.09 | 2.00 | 1.91 |
| 1960–64 | 1.69 | 1.74 | 1.79 |  | 1960–64 | 2.07 | 1.98 | 1.88 |
| 1965–67 | 1.61 | 1.71 | 1.75 |  | 1965–69 | 1.97 | 1.93 | 1.87 |
|  |  |  |  |  | 1970–72 | 1.89 | 1.90 | 1.87 |

Table 5. Childlessness (%) for men (at age 45) and women (at age 40) by educational

attainment in five-year cohorts born in each Nordic country 1940–.

| a) Men: Childless at age 45 | | |  |  | b) Women: childless at age 40 | | |  |
| --- | --- | --- | --- | --- | --- | --- | --- | --- |
|  |  |  |  |  |  |  |  |  |
| Denmark |  |  |  |  | Denmark |  |  |  |
|  | Education |  |  |  |  | Education |  |  |
| Cohort | Low | Medium | High |  | Cohort | Low | Medium | High |
| 1940–44 | 21 | 12 | 12 |  | 1940–44 | 8 | 9 | 12 |
| 1945–49 | 23 | 15 | 16 |  | 1945–49 | 9 | 9 | 13 |
| 1940–54 | 28 | 20 | 19 |  | 1950–54 | 11 | 11 | 15 |
| 1955–59 | 30 | 22 | 21 |  | 1955–59 | 13 | 14 | 16 |
| 1960–64 | 32 | 21 | 20 |  | 1960–64 | 15 | 13 | 15 |
| 1965–69 | 34 | 22 | 20 |  | 1965–69 | 17 | 12 | 14 |
|  |  |  |  |  | 1970–74 | 19 | 13 | 14 |
|  |  |  |  |  |  |  |  |  |
| Finland |  |  |  |  | Finland |  |  |  |
|  | Education |  |  |  |  | Education |  |  |
| Cohort | Low | Medium | High |  | Cohort | Low | Medium | High |
| 1940–44 | 23 | 19 | 11 |  | 1940–44 | 12 | 14 | 21 |
| 1945–49 | 24 | 20 | 13 |  | 1945–49 | 12 | 14 | 19 |
| 1950–54 | 28 | 23 | 16 |  | 1950–54 | 14 | 15 | 20 |
| 1955–59 | 31 | 25 | 18 |  | 1955–59 | 16 | 16 | 19 |
| 1960–64 | 33 | 26 | 21 |  | 1960–64 | 19 | 17 | 20 |
| 1965–67 | 36 | 29 | 22 |  | 1965–69 | 21 | 19 | 21 |
|  |  |  |  |  | 1970–72 | 22 | 22 | 22 |
|  |  |  |  |  |  |  |  |  |
| Norway |  |  |  |  | Norway |  |  |  |
|  | Education |  |  |  |  | Education |  |  |
| Cohort | Low | Medium | High |  | Cohort | Low | Medium | High |
| 1940–44 | 21 | 12 | 10 |  | 1940–44 | 10 | 9 | 14 |
| 1945–49 | 21 | 13 | 11 |  | 1945–49 | 9 | 8 | 13 |
| 1950–54 | 22 | 14 | 13 |  | 1950–54 | 10 | 9 | 14 |
| 1955–59 | 25 | 16 | 15 |  | 1955–59 | 11 | 10 | 14 |
| 1960–64 | 25 | 17 | 16 |  | 1960–64 | 12 | 11 | 14 |
| 1965–69 | 27 | 18 | 17 |  | 1965–69 | 13 | 10 | 13 |
|  |  |  |  |  | 1970–74 | 15 | 11 | 13 |
|  |  |  |  |  |  |  |  |  |

| Sweden |  |  |  |  | Sweden |  |  |  |
| --- | --- | --- | --- | --- | --- | --- | --- | --- |
|  | Education |  |  |  |  | Education |  |  |
| Cohort | Low | Medium | High |  | Cohort | Low | Medium | High |
| 1940–44 | 21 | 15 | 15 |  | 1940–44 | 10 | 12 | 16 |
| 1945–49 | 22 | 17 | 17 |  | 1945–49 | 11 | 11 | 15 |
| 1950–54 | 25 | 20 | 18 |  | 1950–54 | 14 | 12 | 16 |
| 1955–59 | 27 | 22 | 19 |  | 1955–59 | 15 | 14 | 16 |
| 1960–64 | 27 | 22 | 20 |  | 1960–64 | 18 | 14 | 16 |
| 1965–67 | 29 | 22 | 20 |  | 1965–69 | 20 | 13 | 14 |
|  |  |  |  |  | 1970–72 | 21 | 14 | 13 |
